# plantDARIO: web based quantitative and qualitative analysis of small RNA-seq data in plants

**DOI:** 10.3389/fpls.2014.00708

**Published:** 2014-12-23

**Authors:** Deblina Patra, Mario Fasold, David Langenberger, Gerhard Steger, Ivo Grosse, Peter F. Stadler

**Affiliations:** ^1^Institut für Informatik, Martin-Luther-Universität Halle-WittenbergHalle (Saale), Germany; ^2^Bioinformatics Group, Department of Computer Science, Interdisciplinary Center for Bioinformatics, University LeipzigLeipzig, Germany; ^3^ecSeq BioinformaticsLeipzig, Germany; ^4^Institut für Pysikalische Biologie, Heinrich-Heine-UniversitätDüsseldorf, Germany; ^5^German Centre for Integrative Biodiversity Research (iDiv) Halle-Jena-LeipzigLeipzig, Germany; ^6^Max Planck Institute for Mathematics in the SciencesLeipzig, Germany; ^7^Fraunhofer Institute for Cell Therapy and ImmunologyLeipzig, Germany; ^8^Department of Theoretical Chemistry of the University of ViennaVienna, Austria; ^9^Center for RNA in Technology and Health, University of CopenhagenFrederiksberg, Denmark; ^10^Santa Fe InstituteSanta Fe, USA

**Keywords:** non-coding RNA, microRNA, snoRNA, tRNA, high-throughput sequencing, expression analysis, ncRNAome

## Abstract

High-throughput sequencing techniques have made it possible to assay an organism's entire repertoire of small non-coding RNAs (ncRNAs) in an efficient and cost-effective manner. The moderate size of small RNA-seq datasets makes it feasible to provide free web services to the research community that provide many basic features of a small RNA-seq analysis, including quality control, read normalization, ncRNA quantification, and the prediction of putative novel ncRNAs. DARIO is one such system that so far has been focussed on animals. Here we introduce an extension of this system to plant short non-coding RNAs (sncRNAs). It includes major modifications to cope with plant-specific sncRNA processing. The current version of plantDARIO covers analyses of mapping files, small RNA-seq quality control, expression analyses of annotated sncRNAs, including the prediction of novel miRNAs and snoRNAs from unknown expressed loci and expression analyses of user-defined loci. At present *Arabidopsis thaliana*, *Beta vulgaris*, and *Solanum lycopersicum* are covered. The web tool links to a plant specific visualization browser to display the read distribution of the analyzed sample. The easy-to-use platform of plantDARIO quantifies RNA expression of annotated sncRNAs from different sncRNA databases together with new sncRNAs, annotated by our group. The plantDARIO website can be accessed at http://plantdario.bioinf.uni-leipzig.de/.

## 1. Introduction

Plant sncRNAs from seedlings and the inflorescences have been shown to have a broad range of biological functions in the model plant *Arabidopsis thaliana* (Lu et al., 2005). The universe of plant sncRNAs is much more complex and diverse than its counterpart in animals. Longer, approximately or perfectly double-stranded RNA (dsRNA) precursors are cut by Dicer-like (DCL) proteins into small RNA duplexes (Axtell, 2013). The precursors of siRNAs consist of dsRNA molecules (see Bologna and Voinnet, 2014 for a recent review) rather than more or less heavily structured single-stranded RNAs that serve as the precursors of microRNAs (Liu et al., 2014). The small RNA duplexes can be loaded onto different classes of Argonaute (AGO) proteins present in complexes of different functions that mediate the interaction of the incorporated small RNAs with their targets. For e.g., AGO1 acts mainly in microRNA (miRNA) pathways for post-transcriptional gene silencing (PTGS) (Wang et al., 2011a). In case of miRNA duplexes, while the guide strands are incorporated into AGO1 of the RNA-induced silencing complex (RISC), the passenger strands called miRNA star (miRNA^*^) are mostly degraded (Wang et al., 2011b). Small RNAs loaded onto other Argonaute-containing complexes have different functions, e.g., heterochromatin maintenance.

In animals, detailed analyses of small RNA-seq samples, which were primarily produced with the aim of measuring miRNA expression (Hafner et al., 2008; Creighton et al., 2009), revealed that small, roughly microRNA-sized products, are derived from virtually all of the housekeeping ncRNAs including tRNAs (Lee et al., 2009; Sobala and Hutvagner, 2011), snoRNAs (Ender et al., 2008; Falaleeva and Stamm, 2013), and snRNAs (Langenberger et al., 2010; Li et al., 2012b), as well as from many previously undescribed genomic loci including promoters and transcriptional termini of most protein-coding genes (Kapranov et al., 2007). In plants, even more extensive groups of sncRNAs have been described, comprising in addition a variety of distinct types of small interfering RNAs (siRNAs) such as trans-acting siRNAs (ta-siRNAs), natural antisense siRNAs (nat-siRNAs), and double-strand break interacting RNAs (diRNAs) (Mallory and Vaucheret, 2006; Ramachandran and Chen, 2008; Wei et al., 2012; Yoshikawa, 2013). Heterochromatic (hc-)siRNAs are the most abundant class of small RNAs in many plants. The transcripts yielding hc-siRNAs are transcribed by the plant-specific RNA polymerase IV and enter the RNA-directed DNA methylation (RdDM) pathway, comprising first the synthesis of dsRNA by RDR2 and subsequent cleavage by DCL3. The resulting 24 nt long hc-siRNAs are then bound to AGO4 (Matzke and Mosher, 2014). In contrast to miRNAs whose genomic loci are conserved between species, hc-siRNAs genomic loci are not, because they overlap with transposable elements (TEs), which are known to rapidly change their position and copy number in the genomes during plant evolution (Axtell, 2013).

The advent of protocols for preparing small RNA libraries and subsequently sequencing these using Next-Generation Sequencing (NGS) leads to a deluge of small RNA-seq datasets. For the analysis of these RNA-seq data, a large array of computational tools has been developed and published. Most tools focus on the prediction and quantification of sncRNA genes, like ShortStack (Allen et al., 2013), mirDeep (Friedländer et al., 2008), miRanalyzer (Hackenberg et al., 2009), CPSS (Zhang et al., 2012), miRNAkey (Ronen et al., 2010), and omiRas (Müller et al., 2013). Tools such as PsRobot (Wu et al., 2012) combine plant small RNA annotation and target analysis, while psRNATarget (Dai and Zhao, 2011) and SoMART (Li et al., 2012a) are mostly concerned with target prediction. miRanalyzer and omiRas are the only web tools that allow the upload of raw small RNA-seq data in fastq format, while for CPSS and PsRobot the data needs to be formatted to fasta format manually. The other sncRNA prediction tools need to be downloaded, installed and run locally, requiring more than basic computer skills. A drawback of all these tools are the integrated adapter clipping and read mapping steps. Although convenient, this can be problematic since different library preparations and sequencing runs result in sequencing data that should be handled independently. Given the differences in the performance of read mappers, in particular regarding sequences mapping multiple times and the handling of mismatches arising from polymorphisms (Zorc et al., 2012) or editing (Alon et al., 2012), it is desirable, to empower the researcher to use the tools of his/her choice. Furthermore, the sheer size of the raw sequencing data (several gigabyte) compared to their mapping coordinates (some megabyte) and abundances suggests the conclusion, that for a web-tool mapping coordinates are the upload format of choice.

In 2011, DARIO a web server for the analysis of small RNA-seq data in animals was introduced (Fasold et al., 2011). It was designed to perform quality control of input samples, expression analyses of annotated and user-defined sncRNAs, as well as a prediction of new non-coding RNAs. It provides exploratory analyses for mapped, but unannotated reads. Here we present a modified version of this versatile web service specifically tailored to plants. The differences between animal and plant sncRNAs (Bologna et al., 2013) resulted in several modifications in the workflow. Plant pre-miRNAs are much more heterogeneous than their animal counterparts and have a different distribution of genomics contexts in which they reside (Axtell, 2004; Carthew and Sontheimer, 2009; Kim et al., 2009). Hence they are more difficult to annotate (Coruh et al., 2014). In contrast to most animals, plant genomes (with the exception of *Arabidopsis thaliana*) are poorly annotated for ncRNAs and thus a careful and manual annotation of their sncRNAs was essential. A classification of different sncRNAs solely based in their read patterns, as it has been used in DARIO (Fasold et al., 2011), was not possible in plants. Hence, plantDARIO uses third-party tools that also consider sequence and structure information for their predictions. Furthermore, due to a lack of one genome browser covering all plants, it was necessary to adapt and utilize different ones, allowing the researcher to take a look on the read distribution of the known and newly predicted sncRNAs.

## 2. Materials and methods

The current version of plantDARIO handles data for *A. thaliana* (TAIR9 and TAIR10)^1^, *B. vulgaris* (RefBeet-1.1)^2^ (Dohm et al., 2014), and *S. lycopersicum* (SL2.40)^3^ (Tomato Genome Consortium, 2012), and we plan to extend the service to include most of the available plant genomes.

### 2.1. Workflow

The user input to the plantDARIO web service is a list of sequencing read positions mapped to one of the supported reference genomes. Data originating from any sequencing platform and mapped with the user's read alignment tool of choice can be used. However, only data originating from experiments prepared with the small RNA-seq protocol and thus predominantly covering read lengths of about 21–26 nt can be analyzed. Mapped reads can be uploaded in either BAM or bed format. We provide the PERL script map2bed.pl for converting mapped reads to bed format and for merging reads to tags, unique reads. These are represented as coordinate pairs rather than sequences for upload. This reduces the volume of data to be transferred over the internet to a managable amount: 1 GB of SAM formatted mapper output is converted to about 15 MB of compressed bed file that can be uploaded to plantDARIO. User-defined annotations can easily be added to the annotation information stored in plantDARIO's internal database by uploading a list of loci, again in bed format.

**Figure 1:**
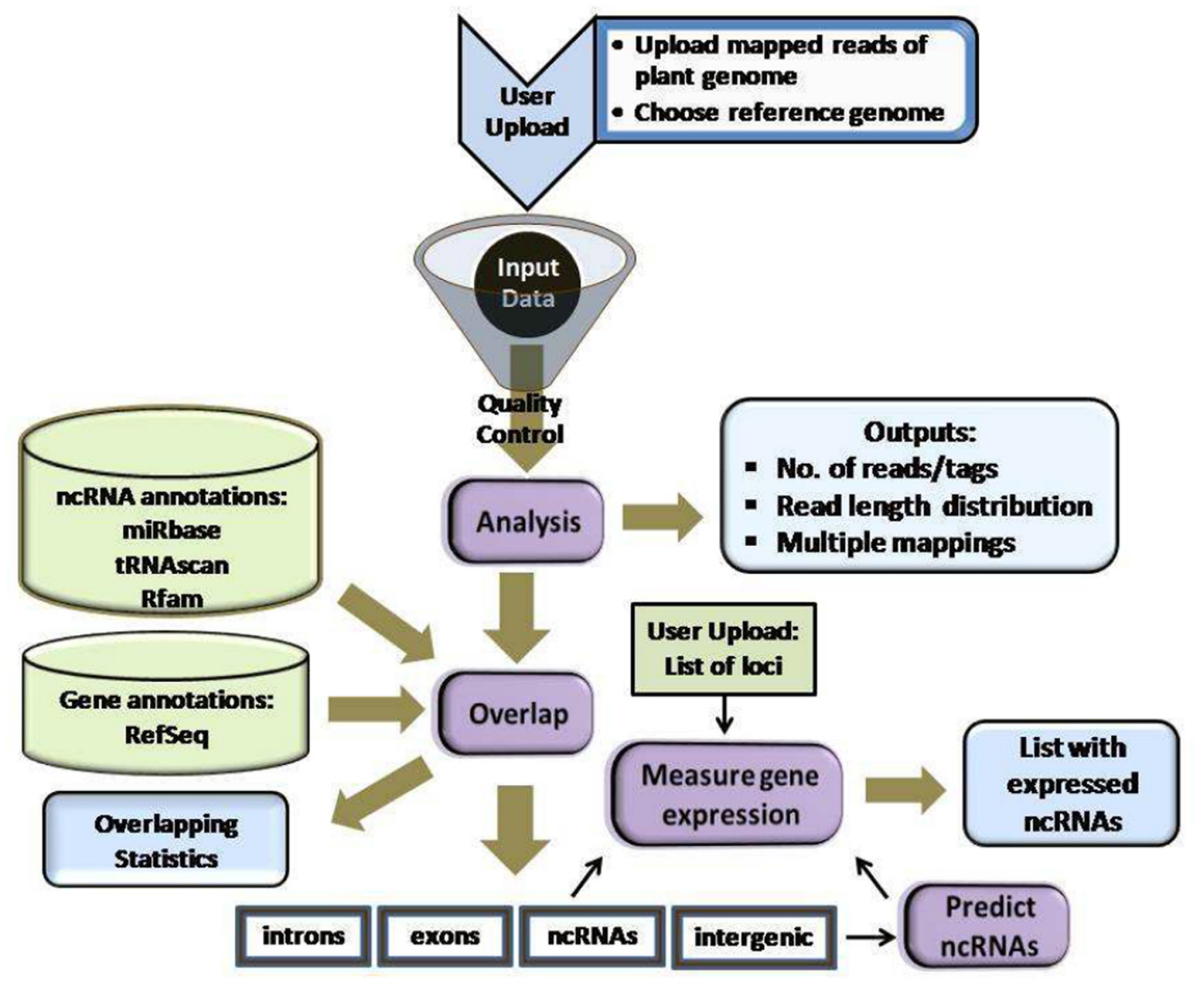
**Workflow design of plantDARIO**. Several analyses are integrated into one step e.g., quantification, normalization processes are merged into the step “Measure gene expression.”

Figure 1 summarizes plantDARIO's workflow, which is similar to that of its animal cousin (Fasold et al., 2011). The usage of plantDARIO is deliberately very similar to its animal cousin and detailed on the separate help page http://plantdario.bioinf.uni-leipzig.de/help.py. Instead of featuring a big extensive pipeline in the workflow, we have collated several analytical works as one step in the workflow. The first component of the pipeline performs a global statistical analysis of the input and provides the aggregate data for several quality control tools. The second component is concerned with the quantitative expression analysis of known and user-defined loci. The third component supports the discovery of novel miRNAs, snoRNAs, and tRNA-like loci. Output is displayed as HTML web pages and provided as machine-readable text files for download. A single job typically takes between 1 and 2 h.

### 2.2. Quality control

A wide variety of errors and biases have been described in high-throughput sequencing data, which may originate from sample handling, library preparation, or the sequencing itself. It is thus necessary to assess the quality and integrity of the experimental data before they are analyzed for biological content (Dohm et al., 2008; Linsen et al., 2009; Hansen et al., 2010). Important measures include the number of mappable reads and the number of tags (distinct read sequences), the distribution of read length, and the sequence composition of mapped reads.

**Figure 2:**
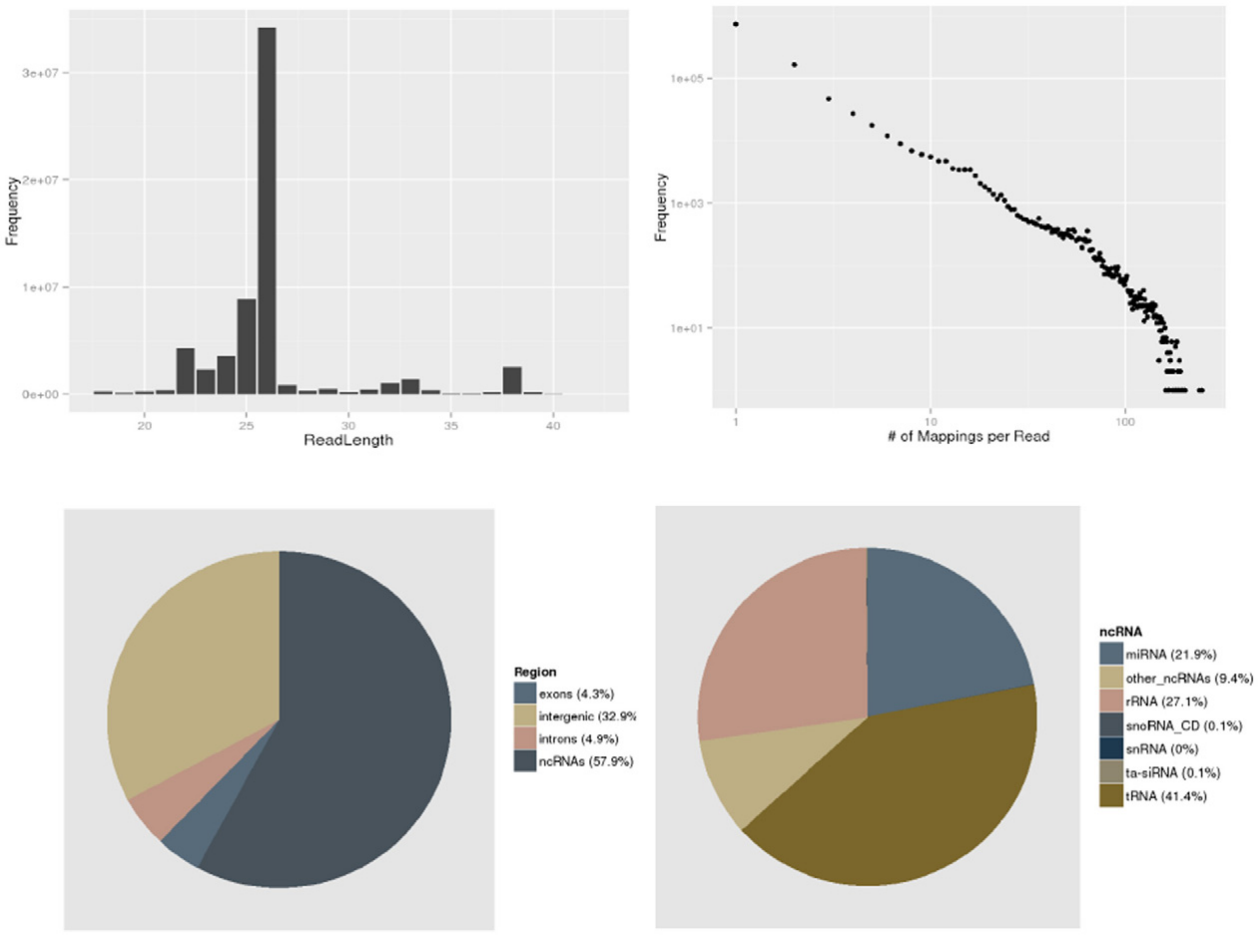
**Initial quality control**. plantDARIO provides overviews of the read length distribution, the distribution of read-length multiplicities, the distribution of genomic locations, and known annotations (separated into known ncRNAs, exons, introns, and intergenic regions). Here, an overview of dataset SRR952330 from *A. thaliana* is shown as an example.

A set of plots provides a convenient overview of the dataset (Figure 2). plantDARIO also computes a summary of the distribution of reads among annotation items such as introns and exons and the major classes of annotated non-coding RNAs such as miRNA, snRNA, rRNA, tRNA, ta-siRNA, and snoRNAs.

### 2.3. RNA quantification

Mapping loci are overlapped with annotated ncRNAs. To this end, plantDARIO includes an internal database of ncRNAs comprising miRNAs from miRBase (Kozomara and Griffiths-Jones, 2011), tRNA annotations from tRNAscan-SE (Lowe and Eddy, 1997), ta-siRNA annotations from TAIR ftp://ftp.arabidopsis.org and tasiRNAdb http://bioinfo.jit.edu.cn/tasiRNADatabase/ (Zhang et al., 2014), plant specific literature data (Barneche et al., 2001; Brown et al., 2001; Dohm et al., 2014), as well as dedicated homology-based annotations for each individual genome. This internal annotation can be complemented by user-defined loci, which are then fully included in all downstream analyses. To handle multiple mappings, the number of reads for each sequence tag is divided by the number of its mapping loci, and this normalized expression value is assigned to each mapping locus.

**Figure 3:**
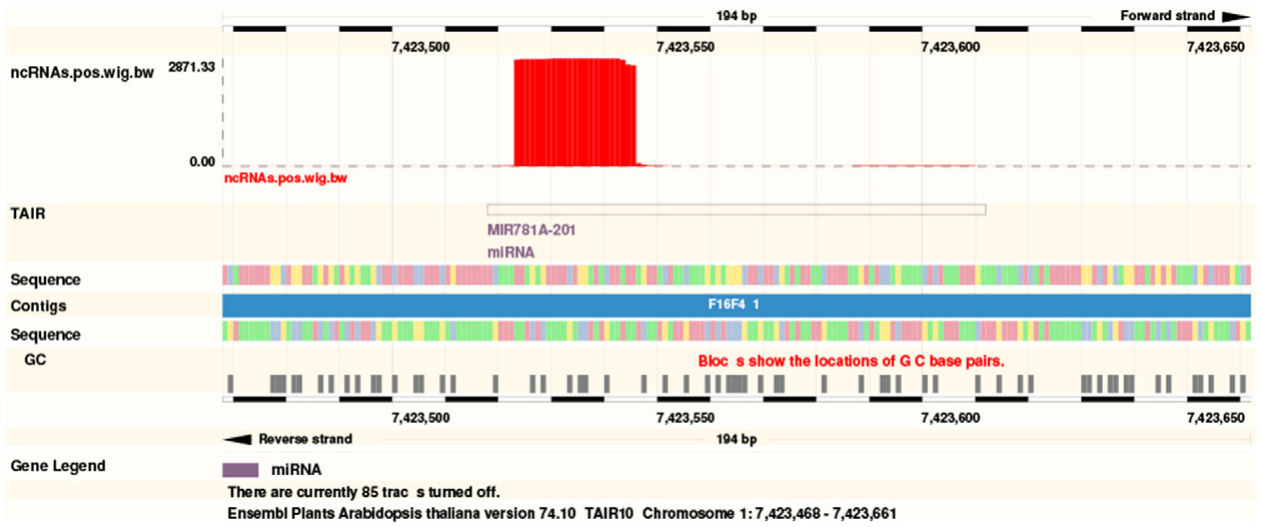
**A link to the Ensemble genome browser (****http://plants.ensembl.org****) allows the instantaneous inspection of ncRNAs with help of ncRNA annotation tracks and conservation**. The example shows the MIR781A-2.1 locus.

The web server generates a list of expressed ncRNAs, itemized by ncRNA classes. For each of them, a normalized expression value based on RPM (Reads per million) and the number of mapped reads (both in raw form and normalized for multiple mapping) is displayed. In addition a link to a genome browser is generated that allows the user to conveniently inspect the expression pattern at each individual locus (Figure 3). This can be helpful e.g., to distinguish between *bona fide* miRNAs from other RNA classes in case of misannotations (Langenberger et al., 2011), to inspect miRNA genes for the presence of offset RNAs (Langenberger et al., 2009; Shi et al., 2009), or to look for short reads generated from the antisense locus (Stark et al., 2008).

### 2.4. Analysis of unannotated loci

**Figure 4:**
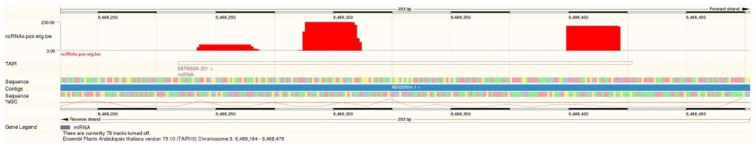
**Usual read patterns of plant microRNAs**. The example shows the MIR868A-201 locus.

Mapped tags are merged to blocks and are aggregated to regions of blocks using blockbuster (Langenberger et al., 2009) with default parameters. Contrary to animals, the processing patterns of miRNAs are not very consistent in plants (Figure 4) so that patterns of mapped reads alone do not allow a sufficiently accurate classification. The same is true for snoRNAs. Hence the prediction of miRNAs and snoRNAs is assisted by the integration of novomir (Teune and Steger, 2010) and snoReport (Hertel et al., 2008) in plantDARIO. These tools are integrated as algorithms or scripts within the plantDARIO software. Both tools implement RNA folding and machine learning approaches to classify intervals of genomic sequences. We use blockbuster to identify accumulations of reads and then run the two tools on these loci.

### 2.5. ncRNA annotation in *Solanum lycopersicum*

Non-coding RNAs have not been comprehensively annotated in many published genomes. This is also the case for *S. lycopersicum*, whereas most relevant annotation data were already available for the arabidopsis and sugar beet genomes. Hence, we produced an annotation track focussing on miRNAs, snoRNAs, and tRNAs for the tomato genome roughly following the workflow employed for the annotation of the *B. vulgaris* genome (Dohm et al., 2014):

1. For miRNAs, plant miRNA pre-cursors were downloaded from miRBase and mapped against the genome using blast, employing a minimum alignment length of 60 nt and a sequence similarity of 80% as filter criteria. Overlapping matches were combined.
2. For snoRNAs, all plant snoRNAs were downloaded from the Rfam database and mapped against the genome with blast, employing a minimum alignment length of 70 nt and a sequence similarity of 80% as filter criteria. Overlapping matches were combined.
3. For tRNAs, tRNAscan (Lowe and Eddy, 1997) was run against the whole genome of *S. lycopersicum*.

The annotations can be downloaded from http://plantdario.bioinf.uni-leipzig.de/annotations/.

### 2.6. snRNA annotation in *Solanum lycopersicum* and *Arabidopsis thaliana*

For the *B. vulgaris* genome, snRNAs are already annotated and available along with other non-coding genes from the *B. vulgaris* resource (Dohm et al., 2014). For *A. thaliana* and *S. lycopersicum*, snRNA covariance models were downloaded from Rfam (ftp://ftp.ebi.ac.uk/pub/databases/Rfam/), and infernal (Nawrocki, 2014) was run against the respective genomes. For the purpose of providing a brief summary statistics, the spliceosomal RNAs U1, U2, U4, U5, U6, U11, U12, U4atac, and U6atac are grouped together with SRP RNA and RNase MRP RNA in the class “snRNAs.” They can be downloaded from the annotation URL given above.

### 2.7. Genomes and visualization

plantDARIO references to the Ensembl genome browser (Hubbard et al., 2002) to visualize the read coverage at annotated loci and predictions as custom tracks for *A. thaliana*. This allows an interpretation of the user data in the context of information provided by the Gramene database (Youens-Clark et al., 2010), a resource for plant comparative genomics. For sugarbeet and tomato, we rely on the genome browser from the *B. vulgaris* resource (Dohm et al., 2014) and sol genomics network (SGN) (Tomato Genome Consortium, 2012), respectively, for visualization.

### 2.8. Implementation details

The technical details of plantDARIO parallel those of DARIO (Fasold et al., 2011). Web pages are created by python scripts making use of the Mako template engine. Graphics are created using R and the graphics package ggplot2 (Wickham, 2009). A queuing system is used to distribute analysis jobs.

## 3. Results and discussion

plantDARIO implements basic workflows for the analysis of small RNA-seq data. It allows the user to obtain a comprehensive overview starting after read mapping. To demonstrate the versatility of plantDARIO we re-analyzed publicly available small RNA-seq datasets from *Arabidopsis* SRR952330, (SRR167709 and SRR167710; Pélissier et al., 2011), sugarbeet (SRR868805) (Dohm et al., 2014), and tomato (SRR786984) (Weiberg et al., 2013). We used segemehl (Hoffmann et al., 2009) with default parameters to map the sequencing data to the respective reference genomes. Unlike many other mapping tools, segemehl has full support for multiple-mapping reads which is very important for small RNA-seq (Otto et al., 2014).

### 3.1. New miRNAs and snoRNAs

**Figure 5:**
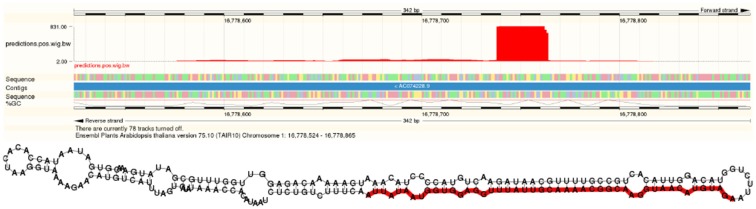
**A novel microRNA discovered by plantDARIO**. **Top** Visualization of the expression profile. **Bottom** Secondary structure of the predicted microRNA precursor.

In addition to more than 200 known miRNAs, we observed more than 100 expressed putative novel miRNAs in each of the datasets (Table 1). An example of a newly predicted miRNA is shown in Figure 5. It represents a perfect plant miRNA pattern as expected for sncRNAs processed by a plant DCL enzyme (Kurihara and Watanabe, 2004), resulting in one functional arm (proper read block in the figure) in this case. The irregular patterns found as little bumps in the structure are bulge loops or internal loops present in the pre-miRNA structure, which are usual, i.e., which are a thermodynamic feature of the RNA. Furthermore, the read pattern matches a stem-loop when traced back to a likely pre-microRNA, as shown in Figure 5.

**Table 1:** **Known and novel sncRNAs in four test datasets**.

		**miRNAs**		**snoRNAs**	
**Data**	**Species**	**Known**	**New**	**Known**	**New**
SRR167709	*A. th*.	276	121	78	348
SRR167710	*A. th*.	236	139	71	268
SRR786984	*S. ly*.	268	65	121	202
SRR868805	*B. vu*.	197	41	60	22

*For both microRNAs and snoRNAs, the number of expressed annotated sncRNA loci (“known”) and the number of novel candidates (“new”) is reported*.

**Figure 6:**
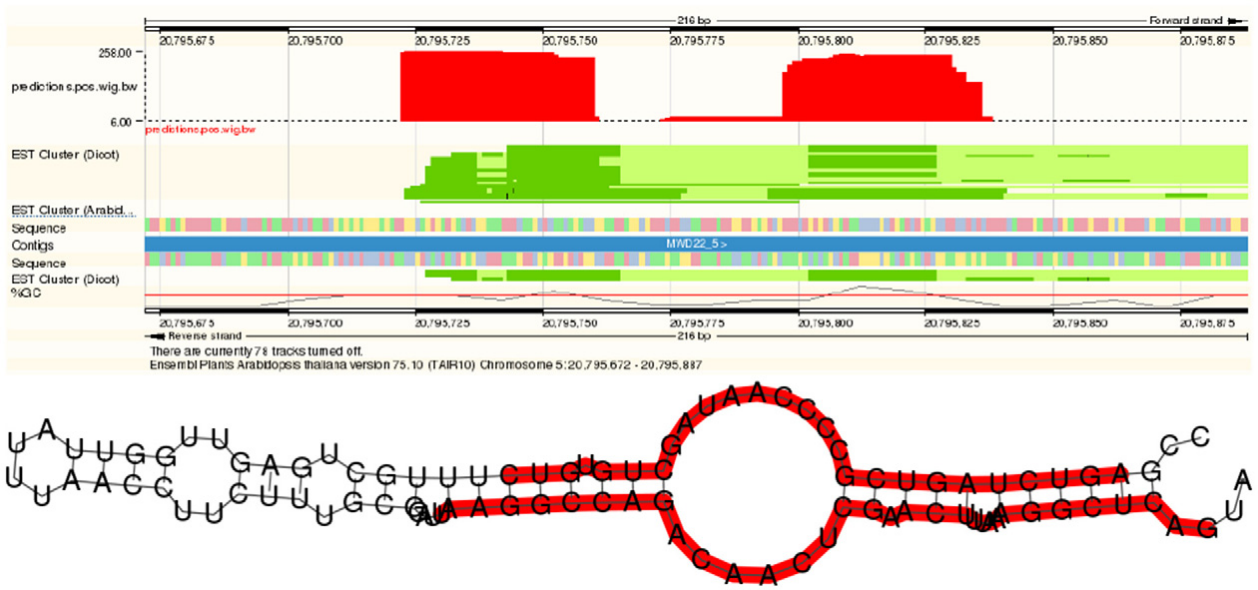
**A novel CD box snoRNA discovered by plantDARIO**. **Top** Visualization of the expression profile. **Bottom** predicted secondary structure; the orgin of the observed short reads is marked in red.

For snoRNAs, we observed an even larger number of candidates. An example is detailed in Figure 6. The structure pattern shows a candidate snoRNA with typical C box and D box sequence patterns close to the ends. The middle region, presumably a loop, contains box C′ and D′ regions frequently found in box C/D snoRNAs.

### 3.2. Differential expression

**Figure 7:**
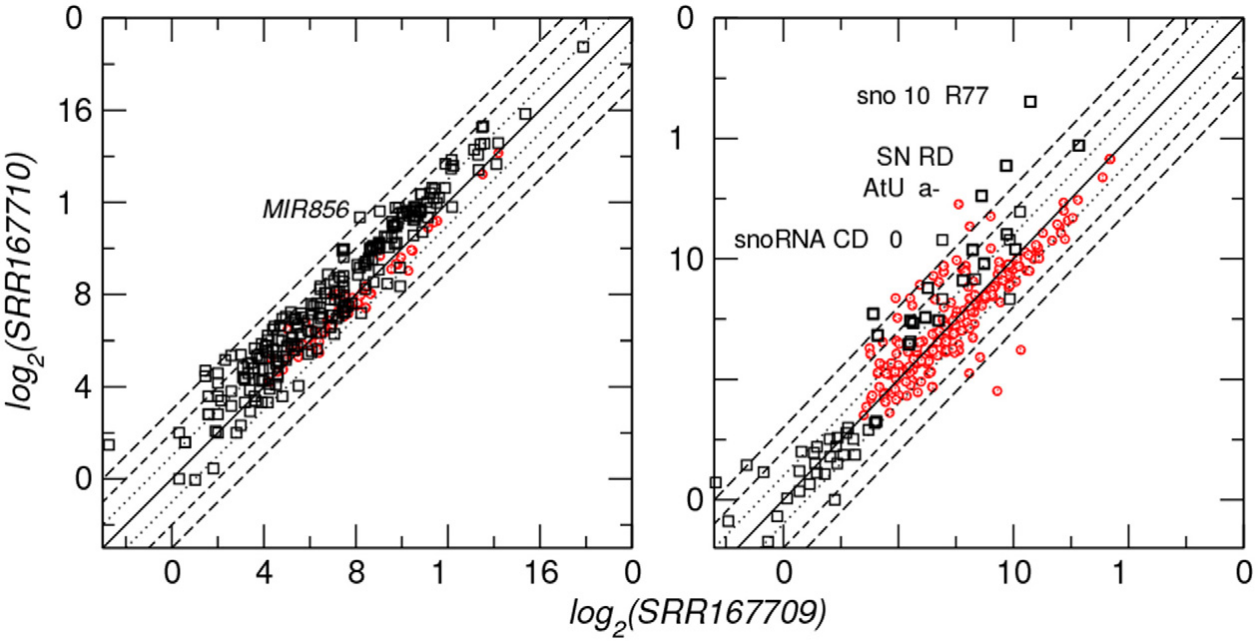
**Differential expression of microRNAs (left panel) and snoRNA-derived small RNAs (right panel) for two *A. thaliana* datasets**. Diagonal lines indicate differences between 2^3^ and 2^−3^ fold. Black symbols indicate annotated microRNA and snoRNA loci, red dots refer to novel predictions. A few loci with extreme expression differences are labeled.

In order to demonstrate that the output of plantDARIO is easy to use for downstream analyses, we compared small RNA expression for miRNA and snoRNA in the two *A. thaliana* datasets SRR167709 and SSR167710 (Pélissier et al., 2011) representing populations of small RNAs from *Arabidopsis* immature flowers of WT and drb2 mutants, respectively. The original study aimed at the antagonistic impact of dsRNA binding proteins DRB2 and DRB4 on polymerase dependent siRNA levels. Figure 7 shows that, overall, the miRNA expression levels correlate positively between the two datasets for both previously annotated and newly predicted miRNAs.

One of the miRNAs with extreme (> 8fold) change in expression level is ath-MIR856. This miRNA, which is predominantly expressed in the floral organ (Meng et al., 2012), belongs to a set of miRNAs that are evolutionary transient within the genus *Arabidopsis* (Ma et al., 2010; Shao et al., 2012) and shows an exceptional evolutionary behavior with relatively low levels of polymorphism but the highest level of divergence (de Meaux et al., 2008).

Surprisingly, we observe a much larger variability for the processing products of snoRNAs. The extreme case, snoZ102_R77, is a box C/D snoRNA belonging to the SNORD44 clan. Box C/D snoRNA_CD_230 (*Arabidopsis*, chr1:6697176-6697261) is related to snoR16 and snoR72 families according to a search in Rfam. All these snoRNAs have a primary function in ribosomal RNA processing (Brown et al., 2003). Interestingly, the examples with extreme differential expression belong to the box C/D class of snoRNAs that is not processed by Dicer but utilizes another, hitherto unknown, processing pathway at least in mammals (Langenberger et al., 2012).

## 4. Concluding remarks

High-throughput sequencing has become the method of choice for the analysis of transcriptome data. For the special case of small RNA-seq data, web services provide a convenient means of conducting standard analyses. In this way the user can avoid the need to install, maintain, and update an array of individual tools. plantDARIO is such a service that, in contrast to comprehensive analysis environments like GALAXY (Goecks et al., 2010), provides a ready-to-use analysis workflow for small RNA-seq data. Together with pre-compiled sncRNA annotations this allows to inspect analysis results quickly after uploading the user data. In summary, plantDARIO provides the user with a valuable combination of annotation-based, standardized quantitative analysis and a simple facility for guided discoveries of novel small RNA loci.

The web service also provides the results in a bed format, which can easily be used for downstream analysis tasks such as the assessment of differential expression. Using publicly available small RNA-seq data for *A. thaliana* we noticed extreme differences in the levels of small RNAs processed from box C/D snoRNAs. Some of these sdRNAs are known to have a regulatory role in animals, so it might be of possible interest to further characterize small RNA processing from “house-keeping ncRNAs” in plants, and plantDARIO might be a convenient and versatile tool for this purpose.

### Conflict of interest statement

The authors declare that the research was conducted in the absence of any commercial or financial relationships that could be construed as a potential conflict of interest.

## Footnotes

^1^ftp://ftp.arabidopsis.org

^2^http://bvseq.molgen.mpg.de

^3^http://solgenomics.net/organism/Solanumlycopersicum/genome

## References

[B1] Allen E. Xie Z. Gustafson A. M. Sung G. H. Spatafora J. W. Carrington J. C. (2013). ShortStack: comprehensive annotation and quantification of small RNA genes. RNA 19, 740–751. 10.1261/rna.035279.112 23610128 PMC3683909

[B2] Alon S. Mor E. Vigneault F. Church G. M. Locatelli F. Galeano F.. (2012). Systematic identification of edited microRNAs in the human brain. Genome Res. 22, 1533–1540. 10.1101/gr.131573.111 22499667 PMC3409266

[B3] Axtell M. J. (2004). Evolution of microRNA genes by inverted duplication of target gene sequences in *Arabidopsis thaliana*. Nat. Genet. 36, 1282–1290. 10.1038/ng1478 15565108

[B4] Axtell M. J. (2013). Classification and comparison of small RNAs from plants. Annu. Rev. Plant Biol. 64, 137–159. 10.1146/annurev-arplant-050312-120043 23330790

[B5] Barneche F. Gaspin C. Guyot R. Echeverria M. (2001). Identification of 66 box c/d snornas in *Arabidopsis thaliana*: extensive gene duplications generated multiple isoforms predicting new ribosomal RNA 2′-o-methylation sites. J. Mol. Biol. 1, 57–73. 10.1006/jmbi.2001.4851 11469857

[B6] Bologna N. Voinnet O. (2014). The diversity, biogenesis, and activities of endogenous silencing small RNAs in arabidopsis. Annu. Rev. Plant Biol. 65, 473–503. 10.1146/annurev-arplant-050213-035728 24579988

[B7] Bologna N. G. Schapire A. L. Palatnik J. F. (2013). Processing of plant microRNA precursors. Brief. Funct. Genomics 12, 37–45. 10.1093/bfgp/els050 23148323

[B8] Brown J. Clark G. Leader D. Simpson C. Lowe T. (2001). Multiple snoRNA gene clusters from *Arabidopsis*. RNA 12, 1817–1832. 10.1017/S1355838201011980 11780637 PMC1370220

[B9] Brown J. W. Echeverria M. Qu L. H. Lowe T. M. Bachellerie J. P. Hüttenhofer A.. (2003). Plant snoRNA database. Nucleic Acids Res. 31, 432–435. 10.1093/nar/gkg009 12520043 PMC165456

[B10] Carthew R. W. Sontheimer E. J. (2009). Origins and mechanisms of miRNAs and siRNAs. Cell 136, 642–655. 10.1016/j.cell.2009.01.035 19239886 PMC2675692

[B11] Coruh C. Shahid S. Axtell M. (2014). Seeing the forest for the trees: annotating small RNA producing genes in plants. Curr. Opin. Plant Biol. 18, 87–95. 10.1016/j.pbi.2014.02.008 24632306 PMC4001702

[B12] Creighton C. J. Reid J. G. Gunaratne P. H. (2009). Expression profiling of microRNAs by deep sequencing. Brief. Bioinform. 10, 490–497. 10.1093/bib/bbp019 19332473 PMC2733187

[B13] Dai X. Zhao P. X. (2011). psRNATarget: a plant small RNA target analysis server. Nucleic Acids Res. 39, W155–W159. 10.1093/nar/gkr319 21622958 PMC3125753

[B14] de Meaux J. Hu J. Y. Tartler U. Goebel U. (2008). Structurally different alleles of the ath-MIR824 microRNA precursor are maintained at high frequency in *Arabidopsis thaliana*. Proc. Natl. Acad. Sci. U.S.A 26, 8994–8999. 10.1073/pnas.0803218105 18579782 PMC2440359

[B15] Dohm J. Minoche A. Holtgräwe D. Capella-Gutiérrez S. Zakrzewski F. Tafer H.. (2014). The genome of the recently domesticated crop plant sugar beet *Beta vulgaris*. Nature 7484, 546–549. 10.1038/nature12817 24352233

[B16] Dohm J. C. Lottaz C. Borodina T. Himmelbauer H. (2008). Substantial biases in ultra-short read data sets from high-throughput DNA sequencing. Nucleic Acids Res. 36:e105. 10.1093/nar/gkn425 18660515 PMC2532726

[B17] Ender C. Krek A. Friedländer M. R. Beitzinger M. Weinmann L. Chen W.. (2008). A human snoRNA with microRNA-like functions. Mol. Cell 32, 519–528. 10.1016/j.molcel.2008.10.017 19026782

[B18] Falaleeva M. Stamm S. (2013). Processing of snoRNAs as a new source of regulatory non-coding RNAs: snoRNA fragments form a new class of functional RNAs. Bioessays 35, 46–54. 10.1002/bies.201200117 23180440 PMC3732821

[B19] Fasold M. Langenberger D. Binder H. Stadler P. F. Hoffmann S. (2011). DARIO: a ncRNA detection and analysis tool for next-generation sequencing experiments. Nucleic Acids Res. 39, W112–W117. 10.1093/nar/gkr357 21622957 PMC3125765

[B20] Friedländer M. R. Chen W. Adamidi C. Maaskola J. Einspanier R. Knespel S.. (2008). Discovering microRNAs from deep sequencing data using miRDeep. Nat. Biotechnol. 26, 407–415. 10.1038/nbt1394 18392026

[B21] Goecks J. Nekrutenko A. Taylor J. The Galaxy Team (2010). Galaxy: a comprehensive approach for supporting accessible, reproducible, and transparent computational research in the life sciences. Genome Biol. 11:R86. 10.1186/gb-2010-11-8-r86 20738864 PMC2945788

[B22] Hackenberg M. Sturm M. Langenberger D. Falcon-Perez J. M. Aransay A. M. (2009). miRanalyzer: a microRNA detection and analysis tool for next-generation sequencing experiments. Nucleic Acids Res. 37, W68–W76. 10.1093/nar/gkp347 19433510 PMC2703919

[B23] Hafner M. Landgraf P. Ludwig J. Rice A. Ojo T. Lin C.. (2008). Identification of microRNAs and other small regulatory RNAs using cDNA library sequencing. Methods 44, 3–12. 10.1016/j.ymeth.2007.09.009 18158127 PMC2847350

[B24] Hansen K. D. Brenner S. E. Dudoit S. (2010). Biases in Illumina transcriptome sequencing caused by random hexamer priming. Nucleic Acids Res. 38, e131. 10.1093/nar/gkq224 20395217 PMC2896536

[B25] Hertel J. Hofacker I. Stadler P. (2008). SnoReport: computational identification of snoRNAs with unknown targets. Bioinformatics 24, 158–164. 10.1093/bioinformatics/btm464 17895272

[B26] Hoffmann S. Otto C. Kurtz S. Sharma C. Khaitovich P. Vogel J.. (2009). Fast mapping of short sequences with mismatches, insertions and deletions using index structures. PLoS Comp. Biol. 5:e1000502. 10.1371/journal.pcbi.1000502 19750212 PMC2730575

[B27] Hubbard T. Barker D. Birney E. Cameron G. Chen Y. Clark L.. (2002). The Ensembl genome database project. Nucleic Acids Res. 30, 38–41. 10.1093/nar/30.1.38 11752248 PMC99161

[B28] Kapranov P. Cheng J. Dike S. Nix D. Duttagupta R. Willingham A. T.. (2007). RNA maps reveal new RNA classes and a possible function for pervasive transcription. Science 316, 1484–1488. 10.1126/science.1138341 17510325

[B29] Kim V. N. Han J. Siomi M. C. (2009). Biogenesis of small RNAs in animals. Nat. Rev. Mol. Cell Biol. 10, 126–139. 10.1038/nrm2632 19165215

[B30] Kozomara A. Griffiths-Jones S. (2011). miRBase: integrating microRNA annotation and deep-sequencing data. Nucleic Acids Res. 39, D152–D157. 10.1093/nar/gkq1027 21037258 PMC3013655

[B31] Kurihara Y. Watanabe Y. (2004). Arabidopsis micro-RNA biogenesis through Dicer-like 1 protein functions. Proc. Natl. Acad. Sci. U.S.A 101, 12753–12758. 10.1073/pnas.0403115101 15314213 PMC515125

[B32] Langenberger D. Bartschat S. Hertel J. Hoffmann S. Tafer H. Stadler P. F. (2011). MicroRNA or not MicroRNA? in Advances in Bioinformatics and Computational Biology, 6th Brazilian Symposium on Bioinformatics, BSB 2011, Vol. 6832 of Lecture Notes in Computer Science, eds de Souza O. N. Telles G. P. Palakal M. J. (Berlin; Heidelberg: Springer), 1–9.

[B33] Langenberger D. Bermudez-Santana C. Hertel J. Hoffmann S. Khaitovich S. Stadler P. F. (2009). Evidence for human microRNA-offset RNAs in small RNA sequencing data. Bioinformatics 25, 2298–2301. 10.1093/bioinformatics/btp419 19584066

[B34] Langenberger D. Bermudez-Santana C. Stadler P. F. Hoffmann S. (2010). Identification and classification of small RNAs in transcriptome sequence data. Pac. Symp. Biocomput. 15, 80–87. 10.1142/9789814295291_0010 19908360

[B35] Langenberger D. Çakir M. V. Hoffmann S. Stadler P. F. (2012). Dicer-processed small RNAs: rules and exceptions. J. Exp. Zool. B Mol. Dev. Evol. 320, 35–46. 10.1002/jez.b.22481 23165937

[B36] Lee Y. S. Shibata Y. Malhotra A. Dutta A. (2009). A novel class of small RNAs: tRNA-derived RNA fragments (tRFs). Genes Dev. 23, 2639–2649. 10.1101/gad.1837609 19933153 PMC2779758

[B37] Li F. Orban R. Baker B. (2012a). Somart: a webserver for plant mirna, tasirna and target gene analysis. Plant J. 70, 891–901. 10.1111/j.1365-313X.2012.04922.x 22268718

[B38] Li Z. Ender C. Meister G. Moore P. S. Chang Y. John B. (2012b). Extensive terminal and asymmetric processing of small RNAs from rRNAs, snoRNAs, snRNAs, and tRNAs. Nucleic Acids Res. 40, 6787–6799. 10.1093/nar/gks307 22492706 PMC3413118

[B39] Linsen S. E. deWit E. Janssens G. Heater S. Chapman L. Parkin R. K.. (2009). Limitations and possibilities of small RNA digital gene expression profiling. Nat. Methods 6, 474–476. 10.1038/nmeth0709-474 19564845

[B40] Liu Y. Wang M. Wang X. (2014). Endogenous small RNA clusters in plants. Genomics Proteomics Bioinformatics 12, 64–71. 10.1016/j.gpb.2014.04.003 24769055 PMC4411336

[B41] Lowe T. Eddy S. (1997). tRNAscan-SE: a program for improved detection of transfer RNA genes in genomic sequence. Nucl. Acids Res. 25, 955–964. 10.1093/nar/25.5.0955 9023104 PMC146525

[B42] Lu C. Tej S. S. Luo S. Haudenschild C. Meyers B. C. Green P. J. (2005). Elucidation of the small RNA component of the transcriptome. Science 309, 1567–1569. 10.1126/science.1114112 16141074

[B43] Ma Z. Coruh C. Axtell M. J. (2010). *Arabidopsis lyrata* small RNAs: transient MIRNA and small interfering RNA loci within *Arabidopsis* genus. Plant Cell 22, 1090–1103. 10.1105/tpc.110.073882 20407023 PMC2879747

[B44] Mallory A. C. Vaucheret H. (2006). Functions of microRNAs and related small RNAs in plants. Nat. Genet. 38, S31–S36. 10.1038/ng1791 16736022

[B45] Matzke M. A. Mosher R. A. (2014). RNA-directed DNA methylation: an epigenetic pathway of increasing complexity. Nat. Rev. Genet. 6, 394–408. 10.1038/nrg3683 24805120

[B46] Meng Y. Shao C. Ma X. Wang H. Chen M. (2012). Expression-based functional investigation of the organ-specific microRNAs in *Arabidopsis*. PLoS ONE 11:e50870. 10.1371/journal.pone.0050870 23226412 PMC3511311

[B47] Müller S. Rycak L. Winter P. Kahl G. Koch I. Rotter B. (2013). omiRas: a web server for differential expression analysis of miRNAs derived from small RNA-Seq data. Bioinformatics 29, 2651–2652. 10.1093/bioinformatics/btt457 23946503

[B48] Nawrocki E. (2014). Annotating functional RNAs in genomes using infernal. Methods Mol. Biol. 1097, 163–197. 10.1007/978-1-62703-709-9-9 24639160

[B49] Otto C. Stadler P. Hoffmann S. (2014). Lacking alignments? the next-generation sequencing mapper segemehl revisited. Bioinformatics 30, 1837–1843. 10.1093/bioinformatics/btu146 24626854

[B50] Pélissier T. Clavel M. Chaparro C. Pouch-Pélissier M. N. Vaucheret H. Deragon J. M. (2011). Double-stranded RNA binding proteins DRB2 and DRB4 have an antagonistic impact on polymerase IV-dependent siRNA levels in *Arabidopsis*. RNA 17, 1502–1510. 10.1261/rna.2680711 21700726 PMC3153974

[B51] Ramachandran V. Chen X. (2008). Small RNA metabolism in *Arabidopsis*. Trends Plant Sci. 13, 368–374. 10.1016/j.tplants.2008.03.008 18501663 PMC2569976

[B52] Ronen R. Gan I. Modai S. Sukacheov A. Dror G. Halperin E.. (2010). miRNAkey: a software for microRNA deep sequencing analysis. Bioinformatics 26, 2615–2616. 10.1093/bioinformatics/btq493 20801911

[B53] Shao C. Ma X. Chen M. Meng Y. (2012). Characterization of expression patterns of small RNAs among various organs in *Arabidopsis* and rice based on 454 platform generated high throughput sequencing data. Plant Omics J. 3, 298–304 10.1016/j.gene.2012.11.015

[B54] Shi W. Hendrix D. Levine M. Haley B. (2009). A distinct class of small RNAs arises from pre-miRNA-proximal regions in a simple chordate. Nat. Struct. Mol. Biol. 16, 183–189. 10.1038/nsmb.1536 19151725 PMC2746024

[B55] Sobala A. Hutvagner G. (2011). Transfer RNA-derived fragments: origins, processing, and functions. Wiley Interdiscip. Rev. RNA 2, 853–862. 10.1002/wrna.96 21976287

[B56] Stark A. Bushati N. Jan C. H. Kheradpour P. Hodges E. Brennecke J.. (2008). A single Hox locus in *Drosophila* produces functional microRNAs from opposite DNA strands. Genes Dev. 22, 8–13. 10.1101/gad.1613108 18172160 PMC2151017

[B57] Teune J. H. Steger G. (2010). NOVOMIR: *De Novo* prediction of microRNA-coding regions in a single plant-genome. J. Nucleic Acids 2010:495904. 10.4061/2010/495904 20871826 PMC2943127

[B58] Tomato Genome Consortium (2012). The tomato genome sequence provides insights into fleshy fruit evolution. Nature 485, 635–641. 10.1038/nature11119 22660326 PMC3378239

[B59] Wang H. Zhang X. Liu J. Kiba T. Woo J. Ojo T.. (2011a). Deep sequencing of small RNAs specifically associated with Arabidopsis AGO1 and AGO4 uncovers new AGO functions. Plant J. 67, 292–304. 10.1111/j.1365-313X.2011.04594.x 21457371 PMC3135789

[B60] Wang X. Laurie J. Liu T. Wentz J. Liu X. (2011b). Computational dissection of arabidopsis smRNAome leads to discovery of novel microRNAs and short interfering RNAs associated with transcription start sites. Genomics 97, 235–243. 10.1016/j.ygeno.2011.01.006 21295131 PMC3707929

[B61] Wei W. Ba Z. Gao M. Wu Y. Ma Y. Amiard S.. (2012). A role for small RNAs in DNA double-strand break repair. Cell 149, 101–112. 10.1016/j.cell.2012.03.002 22445173

[B62] Weiberg A. Wang M. Lin F. Zhao H. Zhang Z. Kaloshian I.. (2013). Fungal small RNAs suppress plant immunity by hijacking host RNA interference pathways. Science 342, 118–123. 10.1126/science.1239705 24092744 PMC4096153

[B63] Wickham H. (2009). ggplot2: Elegant Graphics for Data Analysis. New York, NY: Springer 10.1007/978-0-387-98141-3

[B64] Wu H. Ma Y. Chen T. Wang M. Wang X. (2012). PsRobot: a web-based plant small RNA meta-analysis toolbox. Nucleic Acids Res. 40, W22–W28. 10.1093/nar/gks554 22693224 PMC3394341

[B65] Yoshikawa M. (2013). Biogenesis of trans-acting siRNAs, endogenous secondary siRNAs in plants. Genes Genet. Syst. 88, 77–84. 10.1266/ggs.88.77 23832299

[B66] Youens-Clark K. Buckler E. Casstevens T. Chen C. Declerck G. Derwent P.. (2010). Gramene database in 2010: updates and extensions. Nucleic Acids Res. 39, 1085–1094. 10.1093/nar/gkq1148 21076153 PMC3013721

[B67] Zhang C. Li G. Zhu S. Zhang S. Fang J. (2014). tasiRNAdb: a database of ta-siRNA regulatory pathways. Bioinformatics 30, 1045–1046. 10.1093/bioinformatics/btt746 24371150

[B68] Zhang Y. Xu B. Yang Y. Ban R. Zhang H. Jiang X.. (2012). CPSS: a computational platform for the analysis of small RNA deep sequencing data. Bioinformatics 28, 1925–1927. 10.1371/journal.pone.0030737 22576177

[B69] Zorc M. Jevsinek Skok D. Godnic I. Calin G. A. Horvat S. Jiang Z.. (2012). Catalog of microRNA seed polymorphisms in vertebrates. PLoS ONE 7:e30737. 10.1371/journal.pone.0030737 22303453 PMC3267754

